# Triage policy of severe Covid-19 patients: what to do now?

**DOI:** 10.1186/s13613-020-00770-9

**Published:** 2021-01-27

**Authors:** Pieter Depuydt, Bertrand Guidet

**Affiliations:** 1 Department of Intensive Care, Ghent University Hospital, Department of Internal Medicine and Pediatrics, Ghent University, Corneel Heymanslaan 10, 9000 Gent, Belgium; 2 Sorbonne Universités, UPMC University Paris 06, INSERM, UMR_S 1136, Institut Pierre Louis d’Epidémiologie et de Santé Publique, Equipe: épidémiologie hospitalière qualité et organisation des soins, F-75012 Paris, France; 3 Assistance Publique - Hôpitaux de Paris, Hôpital Saint-Antoine, Service de réanimation médicale, F-75012 Paris, France

We have read with great interest the paper of Dr. Tonetti and colleagues in which they describe the response of the critical care departments of the North Italian hospitals of Lombardy, Veneto and Emilia Romagna in the first 2 weeks of the COVID-19 outbreak in their country, relating this to patient outcome data [1]. ICU departments were expanded by 30% creating 'new' ICU beds by conversion of operation theatres, step-down units and coronary care units. In addition, respiratory support of patients admitted to wards also went to include high-flow oxygen therapy, CPAP and non-invasive ventilation.

The difference in mortality rates in patients admitted to ICUs (47%) and those receiving respiratory support outside the ICU (52%) was small, particularly when considering the fact that patients treated outside the ICU were older and had more comorbidities. Although this report states that managing a subset of COVID-19 patients outside the ICU with advanced non-invasive respiratory support is a reasonable option with mortality rates comparable to that of patients admitted to the ICU, some important questions and caveats remain.

Mortality rates in this report are at the higher end of the hitherto published range, which is itself very broad; mortality rates are very difficult to compare between COVID-19 patient cohorts [2] as it is at present unknown to what extent mortality is determined intrinsically by underlying illness and natural history of the disease or modifiable by therapeutic agents and by avoidance or rapid identification and treatment of complications. Moreover, as patients were classified according to the highest level of care they received, it is not clear how outcome in patients who were admitted directly to the ICU compares to that of patients who were managed on the ward and had a delayed ICU admission; we also do not know if there was a difference in mortality between patients admitted to ‘core’ versus ‘new’ ICU beds, and whether and to what extent strain contributed to lowering standards of care (e.g. as reflected in increasing patient-to-nurse ratio).

As the authors classified patients according to the highest level or respiratory support they received, the 50% mortality of patients treated with conventional oxygen or non-invasive respiratory support (as a highest level) needs further analysis. Given the high number of therapeutic limitations in this patient group, it is likely that most of these were considered too ill, or probably too frail to benefit from invasive ventilation and/or ICU admission: it would be interesting to know what criteria were used for this decision-making. The relative decrease of hospitalized COVID-19 patients admitted to the ICU towards the end of the studied period could point to more stringent criteria for selection imposed by increasing stress on ICU capacity, or reflect the evolving insights in the disease process of severe COVID-19, of which the full spectrum and severity of complications and consequences has increasingly become clear [3].

Since the beginning of the COVID-19 pandemic, important progress has been gained in how to treat these patients (with e.g. corticosteroids, enhanced thrombo-prophylaxis, less (empirical and early) antibiotics) [4]. As such, clinical courses in a subsequent wave may run differently, implying different care allocations, and outcome figures are hopefully now better than in the first ‘COVID-19-naïve’ weeks of the pandemic.

**Fig. 1:**
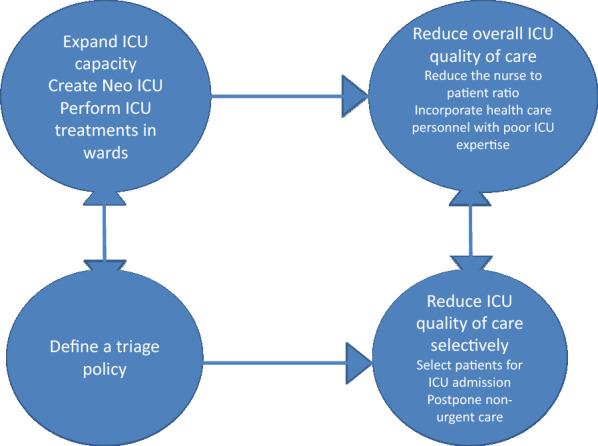
Relation between ICU admission policy, quality of care and critical care organization within the hospital

However, we will still need a global and balanced approach as presented in Fig. 1. A new wave of COVID-19 will probably be more prolonged and coincide with the `flu season` which, regardless of COVID-19, always puts pressure on ICU beds. If we want to apply a pretty liberal ICU admission policy, then we need to expand ICU capacity. The main limiting factor is the availability of health care personnel (HCP) with critical care expertise. Given the shortage of experienced HCP, the nurse-to-patient ratio will become lower and non-experienced nurses will become part of the team. As a result, overall quality of care will diminish probably translating in higher mortality rates. In the opposite, strict admission policy will optimize treatment for ICU patients, but this may come at a cost of diminished chances for patients denied ICU admission [5]. In that perspective, trade-off is unavoidable.
